# Ototoxicity Associated with Antineoplastic Agents in the Pediatric Population: An Evidence-Based Review of Auditory Monitoring Strategies and Contemporary Diagnostic Frameworks—Narrative Review

**DOI:** 10.3390/diagnostics16091272

**Published:** 2026-04-23

**Authors:** Aleksandra Wojno, Oliwia Cichy, Agata Wojno, Karolina Dorobisz, Katarzyna Pazdro-Zastawny

**Affiliations:** 1 Student Scientific Club of Otolaryngoly, Faculty of Medicine, Wroclaw Medical University, 50556 Wroclaw, Poland; oliwia.cichy@student.umw.edu.pl (O.C.); agata.wojno@student.umw.edu.pl (A.W.); 2 Department and Clinic of Otolaryngology, Head and Neck Surgery, Wroclaw Medical University, 50556 Wroclaw, Poland; karolina.dorobisz@umw.edu.pl (K.D.); katarzyna.pazdro-zastawny@umw.edu.pl (K.P.-Z.)

**Keywords:** pediatric oncology, ototoxicity, platinum-based chemotherapy, cisplatin, carboplatin, hearing loss, audiological monitoring, otoacoustic emissions, auditory brainstem response, sodium thiosulfate

## Abstract

Ototoxicity represents a clinically significant complication of anticancer therapy in pediatric patients. Cytotoxic agents used in oncology, particularly platinum-based chemotherapy, may induce damage to the auditory and vestibular systems, resulting in hearing loss, tinnitus, and balance disturbances. Even mild hearing impairment during childhood may negatively affect speech perception, language development, communication abilities, and subsequent educational and psychosocial functioning. This narrative review aims to synthesize current evidence on treatment-related ototoxicity in children, with particular focus on commonly implicated therapies, clinical consequences, diagnostic approaches, and potential preventive strategies. A focused literature search was conducted in PubMed for publications from 2019 to 2025 addressing ototoxicity associated with pediatric anticancer treatment and audiological monitoring methods. The analysis indicates that platinum-based compounds, especially cisplatin and carboplatin, remain the primary agents associated with ototoxicity, with reported incidence ranging from approximately 20–70% for cisplatin and 10–30% for carboplatin. Additional risk factors include young age, baseline hearing status, renal function, and exposure to other ototoxic agents such as aminoglycoside antibiotics. Early detection relies on comprehensive audiological monitoring combining behavioral and objective methods, including pure-tone audiometry, extended high-frequency audiometry, otoacoustic emissions, and auditory brainstem response testing. Standardized grading systems such as ASHA, Brock, Chang, and SIOP Boston criteria play a key role in identifying and classifying ototoxic changes. Emerging research focuses on improved monitoring protocols, biomarker identification, and the development of otoprotective strategies, including sodium thiosulfate and experimental molecular therapies. Implementing systematic hearing monitoring and preventive strategies is essential to reduce long-term auditory complications and improve quality of life in pediatric cancer survivors.

## 1. Introduction

Anticancer treatment in children is primarily based on multi-agent chemotherapy regimens and radiotherapy. Such approaches are associated with a risk of adverse effects resulting from the non-specific action of therapeutic agents on normal cells. A complication of particular clinical relevance and long-term consequences is ototoxicity, defined as a transient or permanent impairment of auditory and/or vestibular function induced by therapeutic substances, manifesting, among others, as hearing loss, tinnitus, and dizziness [1,2].

The consequences of cytotoxicity-related symptoms occurring during treatment are of particular importance, as even mild hearing loss may adversely affect the acquisition of language competencies. More severe high-frequency hearing impairment can disrupt speech perception, speech intelligibility, language development, and communication, which in turn directly translates into subsequent educational and psychosocial functioning [3,4,5]. Ototoxicity may involve not only the cochlea but also vestibular structures, which can additionally lead to balance disturbances and an increased risk of falls and may also lead to delays in motor development [6]. In children, hearing loss may initially remain unnoticed, as it is not always immediately identified in typical communicative situations [3,7]. Therefore, hearing monitoring in pediatric oncology patients should constitute an integral component of management aimed at early detection of auditory disturbances, limiting their progression, and implementing adequate clinical, organizational, and rehabilitative support [2].

Chemotherapy-induced hearing impairment involves three principal sites within the inner ear: the outer hair cells of the organ of Corti, spiral ganglion neurons, and the cells of the stria vascularis [1,6,8,9]. The risk of ototoxicity is influenced by both patient- and treatment-related factors, including, among others, age (particularly very young age), baseline hearing status, renal function, comorbidities, and the concomitant use of other ototoxic medications [1,8,10].

Hearing loss constitutes a growing burden on global public health. According to estimates, it may affect approximately 2.5 billion people worldwide by 2050, of whom at least 700 million will require hearing rehabilitation [11]. From the perspective of low- and middle-income countries, limited access to hearing monitoring remains a major concern. Data from South Africa indicate that ototoxicity monitoring is not routinely implemented, with the main barriers including shortages of personnel and equipment, as well as constraints within referral systems [12,13,14]. The importance of this issue is further underscored by data from Egypt, where hearing loss was identified in 25% of children after treatment with platinum compounds [14].

The aim of this review is not only to synthesize current evidence on treatment-related ototoxicity in children, but also to critically examine the consistency, clinical applicability, and limitations of currently available monitoring strategies and preventive approaches. Specifically, the analysis seeks to: (1) summarize treatment modalities most commonly linked to ototoxicity, with particular emphasis on platinum-based regimens; (2) outline the clinical consequences of hearing impairment during the developmental period; and (3) describe contemporary audiological methods used for the detection and monitoring of ototoxicity, including pure-tone audiometry, high-frequency audiometry, otoacoustic emissions (OAEs), and auditory brainstem response (ABR) testing. In addition, the analysis aims to highlight approaches reported in the literature to mitigate treatment-related ototoxicity and to identify areas requiring further research.

A focused literature search was conducted in PubMed to identify publications addressing ototoxicity associated with pediatric anticancer therapy, its clinical consequences, and audiological methods used for detection and monitoring. The search covered publications from 2019 to 2025 and used combinations of free-text terms related to ototoxicity and hearing outcomes (including “ototoxicity”, “hearing loss”, and “sensorineural hearing loss”), pediatric population (“child”, “children”, “pediatric”), anticancer treatment (“cisplatin”, “carboplatin”, “platinum-based chemotherapy”), and audiological assessment (“pure-tone audiometry”, “high-frequency audiometry”, “otoacoustic emissions”, and “auditory brainstem response”). Boolean operators (AND/OR) were applied to refine the search.

Studies were considered eligible if they addressed pediatric oncology patients and reported data on audiological outcomes, hearing loss, or ototoxicity monitoring. Publications not relevant to pediatric cancer treatment, not focused on ototoxicity or hearing assessment, or lacking clinical/audiological relevance to the topic were excluded. Given the narrative nature of this review, the retrieved literature was selected for relevance and synthesized qualitatively rather than through a formal systematic-review process.

Importantly, the available evidence on pediatric treatment-related ototoxicity remains methodologically heterogeneous, with substantial variation in study design, patient age, cumulative drug exposure or follow-up duration. This limits direct comparability across studies and should be taken into account when interpreting reported incidence rates and proposed monitoring strategies.

## 2. Drugs with Documented Ototoxic Effects

An analysis of data from the FAERS database, comprising reports of adverse events in children aged 0–17 years, demonstrated the presence of significant signals related to hearing impairment for a range of active substances, including anticancer agents. Among the drugs identified, the highest number of reports was recorded for carboplatin and cisplatin [5]. Platinum-based compounds contained in these agents are therefore of particular relevance. The cytotoxic effects of these drugs have been associated, among others, with the induction of oxidative stress and the generation of reactive oxygen species [7,8,15].

Cisplatin (cis-diammine dichloroplatinum(II)) is one of the fundamental anticancer agents, also used in the pediatric population [7,16]. Cisplatin-induced ototoxicity is primarily sensorineural in nature; it is bilateral, progressive, and irreversible, and clinically most commonly presents as hearing loss initially affecting the high-frequency range, often accompanied by tinnitus [4,7,16]. The proportion of patients who develop ototoxicity following cisplatin treatment is estimated to range from approximately 20% to 70% [4,7,9,16]. Younger individuals are particularly vulnerable to hearing loss after cisplatin therapy, and in children under 5 years of age, this impairment may occur already at an early stage of treatment [17].

Cisplatin-induced ototoxicity is closely associated with its distribution to the inner ear and the susceptibility of specific cochlear structures to drug accumulation and the initiation of cytotoxic cascades. The transport of cisplatin into the cochlea largely depends on the barrier separating the bloodstream from the structures of the inner ear. A key component of this barrier is the endothelial cells of cochlear vessels, which are tightly interconnected, thereby limiting the passage of large molecules and blood cells into cochlear tissues [18]. Cisplatin reaches the cochlea primarily via the vascular supply of the stria vascularis and may subsequently appear in the endolymph, which directly bathes the apical surfaces of hair cells [15,19]. This compound exerts its cytotoxic effect through the formation of DNA adducts, which underlies its anticancer efficacy; however, it simultaneously initiates damage cascades in normal tissues, including the inner ear [16]. Moreover, exposure to cisplatin is associated with increased levels of reactive oxygen species (both cellular and mitochondrial) and with the activation of apoptotic pathways in auditory cells [20].

The wide range of reported rates of cisplatin-associated ototoxicity likely reflects both biological variability and important methodological differences across studies. These may be manifested in the use of non-uniform classification systems, differences in drug dosing, patient age, duration of follow-up, and the coexistence of other factors with potential ototoxic effects. Therefore, estimates of incidence should be interpreted with caution.

Carboplatin (CBDCA) is the second key platinum compound used in anticancer therapies. It has been suggested that carboplatin is less ototoxic than cisplatin [21,22]. The proportion of patients affected by ototoxicity following carboplatin therapy has been estimated at approximately 10–30% [22,23,24]. The cytotoxic mechanism of carboplatin, similarly to that of cisplatin, is described as a consequence of damage to inner ear structures and/or the vestibular system, resulting in a characteristic pattern of changes observed on audiometric assessment [23]. Nevertheless, ototoxicity in this context is also considered potentially pharmacologically modifiable, for example, through sodium thiosulfate therapy [25]. Although carboplatin is generally considered less ototoxic than cisplatin, this distinction should not be regarded as direct evidence of a lower risk. The risk associated with carboplatin use may be influenced, among other factors, by concomitant therapeutic modalities, prior exposure to platinum compounds, and differences in the intensity of monitoring.

Aminoglycosides (AGs) constitute a class of broad-spectrum antibiotics used in pediatric practice, including in clinical scenarios typical of oncology patients, such as severe sepsis, septic shock, febrile neutropenia, or infections of potentially multidrug-resistant etiology, where combination therapy with β-lactams may be considered to achieve a synergistic effect [26,27]. Excessive exposure to aminoglycosides may lead to both permanent hearing impairment and vestibular dysfunction [28]. It is indicated that approximately 20% of patients treated with aminoglycosides develop long-term, permanent hearing loss; in some analyses this proportion may reach 50%, while vestibular toxicity has been observed in up to 60% of cases during therapy [29,30]. Importantly, aminoglycoside-related ototoxicity may manifest even after a single intravenous dose, and in populations receiving short courses of therapy (below 16 days), the incidence of hearing loss associated with aminoglycoside exposure has been estimated at 16.6% [31]. Moreover, individual agents within this class differ in their predominant toxicity profile: gentamicin and tobramycin are described as more vestibulotoxic, whereas amikacin is considered more cochleotoxic [31]. Increased susceptibility to aminoglycoside binding is associated with mitochondrial DNA mutations within the 12S rRNA gene, including A1555G and C1494T [26,28].

In the context of novel anticancer therapies with ototoxic potential, special attention should be paid to immune checkpoint inhibitor immunotherapy, as the literature reports audiovestibular adverse events with heterogeneous clinical presentations, including sudden bilateral hearing loss with balance disturbances, milder degrees of hearing impairment, or tinnitus with preserved hearing thresholds [32]. Moreover, therapies not routinely classified as ototoxic may also be associated with late-onset auditory sequelae. Indeed, it has been reported that the proportion of clinically significant hearing loss exceeded 44% in the “late effects” group, whereas in the subgroup treated with methotrexate without exposure to irradiation and without other classical risk factors, hearing loss ranging from mild to profound was identified in 37.5% of patients [33]. At present, the evidence linking newer anticancer therapies and selected non-classical agents to ototoxicity remains more limited than for platinum compounds, and in some cases is based on small series or late-effects cohorts. A summary of key ototoxic agents and recommended audiological monitoring strategies is presented in Table 1.

## 3. Audiological Monitoring: Methods and Grading Criteria

Direct detection of ototoxicity during anticancer therapy requires sensitive diagnostic capable of identifying the earliest auditory changes, before the subjectively perceive communication difficulties. Particularly prior to involvement of the frequency ranges essential for speech perception [14]. Ototoxicity monitoring relies on a complementary use of behavioral and objective assessment methods, which differ both in patient-related demands and in the type of diagnostic information they provide regarding the localization and progression of auditory system impairment. Baseline audiological evaluation should be comprehensive and include pure-tone audiometry in the conventional frequency range, high-frequency audiometry, tympanometry, speech audiometry, and otoacoustic emission testing. Following establishing a reference point, subsequent follow-up assessments should focus on monitoring air-conduction thresholds (PTA), high-frequency audiometry, and otoacoustic emissions [34].

In pediatric patients undergoing anticancer therapy, hearing impairment typically manifest initially in the high-frequency range and may subsequently extend into the frequencies critical for speech perception. This characteristic pattern determines the selection of monitoring tools and the sensitivity of the adopted criteria used to detect auditory disturbances [35,36]. Monitoring based solely on conventional pure-tone audiometry up to 8 kHz may fail to reflect the earliest stages of auditory damage [14,36]. Extended high-frequency (EHF) audiometry, covering the range of 8–20 kHz, demonstrates superior sensitivity in detecting early cochlear injury, as platinum-based chemotherapeutic agents preferentially affect the basal cochlear regions responsible for high-frequency processing. This enables observation of threshold elevation in the EHF range while conventional audiometric thresholds remain within normal limits, before frequencies critical for speech understanding become involved [37]. Despite its superior sensitivity for early cochlear damage, extended high-frequency audiometry is not universally feasible in routine pediatric oncology care, particularly in younger children and in centers with limited equipment or staffing. Its diagnostic value is therefore high, but its implementation may be constrained by real-world organizational factors.

Otoacoustic emissions (OAE) are a valuable complement to the hearing assessment, particularly when increased objectivity is essential and reduce reliance on behavioral responses. OAE testing enables an objective evaluation of outer hair cell function without reliance on patient cooperation, which is particularly useful in young children and in patients whose condition limits the reliability of pure-tone audiometry [34,35,36,37,38]. Otoacoustic emissions may detect changes in cochlear function before a decline becomes evident on the audiogram, thereby making them effective for identifying cochlear dysfunction [2].

Auditory brainstem responses (ABR/BAEP) represent a more conservative objective monitoring approach. These objective methods enable the assessment of auditory function is based on the recording of a bioelectric response that is time-locked to the stimulus [39]. ABR is used to estimate hearing thresholds in infants and young children who are unable to provide reliable behavioral responses [38]. It allows the detection of threshold shifts based on an objective neurophysiological signal, which can be compared with audiometric and otoacoustic emission findings to triangulate conclusions regarding the direction and dynamics of auditory changes [40].

Comparison between subjective and objective monitoring methods indicates that the patient’s perspective is clinically important, yet it may be temporally misaligned with changes detectable through instrumental assessments. This occurs because hearing loss may not be noticed by the patient until communication difficulties become apparent, suggesting that by that time the impairment already involves frequencies essential for speech understanding, i.e., a potentially later stage from the standpoint of preventing further progression of damage [14]. Moreover, auditory changes may be underestimated by patients facing a life-threatening disease, despite their impact on communication and social support, which may contribute to the underdiagnosis and undertreatment of ototoxicity [37]. The key issue is not choosing between subjective and objective monitoring but rather designing a protocol that balances the limitations of each method while remaining feasible to implement within the constraints of oncology care.

It is also critically important to establish organizational frameworks that enable audiological surveillance to be conducted within routine oncological care. Recent reports from the International Ototoxicity Management Group (IOMG) indicate that ototoxicity management should be integrated into existing therapeutic pathways as a structured program based on coordinated care, system-level solutions, and clinical objectives tailored to local conditions [41,42].

The assessment of ototoxicity in studies on anticancer therapies is based on standardized criteria for changes in hearing thresholds. The choice of grading scale is not merely a technical detail, but a major determinant of how ototoxicity is detected, classified, and ultimately reported in the literature. Consequently, differences between studies may reflect not only true clinical variation, but also differences in the diagnostic framework applied [1].

The ASHA criteria are incorporated into ototoxicity assessment primarily in the variant referring to the so-called sensitive range for ototoxicity (SRO), which is based on measurements at 1/6-octave intervals within the highest audible octave for a given patient and is described as capable of identifying over 90% of initial ototoxic threshold shifts [10]. The ASHA criteria primarily serve as a definition of a clinically significant change in hearing thresholds to be detected during monitoring [35]. According to ASHA, a significant threshold change is defined as a ≥20 dB decreases at any single frequency, a ≥10 dB decreases at two adjacent frequencies, or loss of response at the maximum output level of the audiometer at three consecutive frequencies [34].

The Brock scale, in turn, is a classical tool for grading hearing loss associated with exposure to platinum compounds and is characterized by a favorable approach to the classification of high-frequency ototoxicity [1]. It is a pediatric ototoxicity grading system in which clinical categories are constructed based on frequency-dependent thresholds, reflecting the typical high-frequency involvement profile observed in ototoxicity [35]. The successive Brock grades (0–4) refer to the presence of thresholds exceeding 40 dB across an increasingly broad frequency range: from the absence of such thresholds (grade 0), through isolated involvement at 8 kHz (grade 1), then 4–8 kHz (grade 2), 2–8 kHz (grade 3), up to 1–8 kHz (grade 4) [8,35].

As an alternative to classical grading scales, Ehlert et al. applied a classification of hearing loss severity based on the mean pure-tone average (PTA) for 500, 1000, 2000, and 4000 Hz, assigning categories according to the World Health Organization grading system (PTA < 25 dB HL as normal hearing, 26–40 dB HL as mild hearing loss, 41–60 dB HL as moderate, 61–80 dB HL as severe, and > 81 dB HL as profound) [14]. Another approach involves quantifying changes using the ABR threshold and waveform parameters (amplitudes, amplitude ratios, and brainstem conduction time measures), indicating that the classification of changes may take the form of a set of objective indicators differentiating the baseline state from the post-exposure state [39].

Nevertheless, it has been postulated that ototoxicity surveillance should consistently rely on validated assessment systems, such as the SIOP Boston scale, the Chang criteria, or CTCAE [34,35,36,37]. For example, the Chang scale differentiates, among others, situations involving thresholds ≥40 dB in the 6–12 kHz range (grade 1a) and the gradual extension of hearing loss into lower frequency bands, down to thresholds ≥40 dB at 1 kHz and above (grade 4). In contrast, TUNE captures severity based on defined threshold shifts within specific sets of frequencies [35]. With regard to the Boston SIOP method, the procedure begins with the assessment at 4000 Hz, and subsequent frequency selection depends on whether the threshold exceeds 20 dB at this level: if the threshold is >20 dB at 4000 Hz, the next step is to assess 2000 Hz, followed—depending on the result at 2000 Hz—by 1000 Hz or 3000 Hz. If the threshold is <20 dB at 4000 Hz, assessment at 8000 Hz is indicated, and if the threshold is <20 dB at 8000 Hz, additional evaluation at 6000 Hz is recommended [2].

Different classification systems capture different aspects of hearing dysfunction, which substantially limits the comparability of studies. This partly explains why the reported rates of ototoxicity may differ even among similar patient populations.

The implementation of a pediatric monitoring model also requires access to personnel with appropriate expertise and experience. Audiologists are not fully coordinated with all teams providing pediatric oncology care, and this lack of coordination is particularly evident in communication between audiology and oncology [43]. The situation is further complicated by insufficient integration of care, the unmet need for a greater number of specialized personnel, and the lack of fully effective, clinically validated otoprotective interventions [9,16,20,43].

## 4. Clinical Management and Monitoring Algorithms

There is an urgent need to standardize approaches to ototoxicity monitoring in the pediatric population undergoing anticancer treatment [2,7]. An important step was an international consensus meeting on grading ototoxicity, organized during the annual International Society for Pediatric Oncology conference in Boston in 2010, which resulted in the publication of the SIOP scale in 2012 [2,43]. According to these recommendations, clinical management should be based on systematic, comparative hearing assessment conducted at three key time points: prior to exposure, during treatment, and after its completion, in order to capture the dynamics of changes and relate results to baseline value [4]. As a minimum audiological assessment, otoscopic examination, pure-tone audiometry, tympanometry, OAE, and auditory brainstem responses (ABR) are employed, with audiology specialists playing a crucial role in interpreting results and identifying auditory dysfunction [3,4,33,43].

The translation of these principles into an audiological assessment schedule takes the form of a stepwise algorithm comprising baseline evaluation, monitoring during treatment, and post-therapy assessment with follow-up. The minimum standard includes performing a baseline examination within a short window from the initiation of therapy (up to 72 h), followed by systematic repeated assessments throughout treatment, an evaluation at the end of the course, and delayed follow-up visits to detect persistent or late-onset changes [31,44]. After the baseline assessment, evaluation is conducted after each chemotherapy cycle and additionally following potentially further burdening interventions [2,35,37]. The emergence of auditory symptoms (hearing loss, tinnitus) or vestibular symptoms during treatment should prompt consideration of treatment interruption, modification of the therapeutic regimen, and referral for specialist otolaryngological assessment [10,35]. Despite a strong conceptual rationale, monitoring algorithms are associated with substantial challenges in their implementation in routine care. Above all, close coordination between oncology and audiology services is required. Equally important are effective cooperation with children and the availability of adequately trained personnel and appropriate equipment. Therefore, it should be recognized that the effectiveness of a monitoring protocol depends to a large extent on its operational feasibility. The final stage includes an end-of-treatment evaluation and further surveillance, for which annual follow-up visits have been proposed; the minimum test battery includes tympanometry, BAEP, and age-appropriate behavioral audiometry [2,36]. Another approach recommends assessment immediately after treatment and at three- and six-month intervals following therapy completion [45]. It is impossible to overlook the lack of full consensus regarding the optimal interval between assessments. It should therefore be emphasized that follow-up schedules may need to be adapted according to risk.

A parallel component of diagnostic monitoring for ototoxicity in anticancer therapy is prevention and early intervention. It is crucial to conduct a pre-treatment risk assessment, including consideration of coexisting burdens and exposures that may increase the likelihood of hearing deterioration [26,44]. Another key pillar of prevention is limiting exposure to ototoxic agents, including optimizing therapy toward the lowest feasible doses of ototoxic compounds and individualizing treatment accordingly [21,25]. Early interventions using otoprotective measures and systematic hearing monitoring are particularly important, as children constitute one of the most vulnerable groups with respect to ototoxicity, and potential hearing loss may disrupt the critical period of learning as well as the development of social and communicative skills, including language and speech [46]. Therefore, once changes are detected, there is a need for the early implementation of supportive strategies to facilitate the child’s functioning, including hearing assistive technologies (hearing aids, FM systems, cochlear implants), educational and environmental accommodations, communication strategy training, and psychosocial support targeting social difficulties and self-esteem [37].

Regarding otoprotective agents, sodium thiosulfate (STS) warrants particular attention due to its promising potential in clinical settings. Nevertheless, when administered systemically, its potential protective effect on the auditory system may be associated with a risk of reduced anticancer efficacy [29,44]. It should be emphasized that the effect of STS on treatment efficacy must be interpreted in light of disease stage, as confirmed by data from two randomized trials. In the SIOPEL 6 trial, conducted in children with standard-risk hepatoblastoma, STS reduced the incidence of hearing loss from 63% to 33%, with a relative risk of 0.52, corresponding to an approximately 48% relative risk reduction, and was not associated with significant differences in 3-year event-free survival (EFS) or overall survival (OS) between the groups [47,48]. In the second trial (ACCL0431), STS significantly reduced the incidence of hearing loss from 56% to 29%, and in a reanalysis using the SIOP scale, it was associated with a significantly lower risk of clinically significant hearing loss [48,49].

This compound, in an injectable formulation, has been approved by the FDA to reduce the risk of cisplatin-induced ototoxicity in children with localized, non-metastatic solid tumors [20,25]. However, the timing of STS administration relative to cisplatin is of critical importance. In two phase III trials (SIOPEL 6 and COG ACCL0431), STS was administered intravenously as a short infusion initiated 6 h after cisplatin chemotherapy, which was associated with a reduction in the incidence of hearing loss in the pediatric population studied [16]. Other substances reported to have potential protective effects include amifostine, dexamethasone, genistein, Ginkgo biloba, lycopene, N-acetylcysteine, and polydatin [4].

## 5. Overview of Current Scientific Research

An analysis of the available literature reveals the highly dynamic nature of research on ototoxicity, with particular intensification in mechanistic and preclinical areas. Accordingly, while many methods and biomarkers appear promising, their significance has not yet been confirmed for routine clinical use in pediatric oncology. Current research directions in audiology focus on enhancing predictive capabilities, standardizing protocols, improving the objectivity of measurements, and developing data infrastructure that enables multicenter standardization [10]. The digitalization and decentralization of ototoxicity monitoring are of particular importance. Cheong et al. describes a model in which screening assessments are performed on the ward by trained non-audiology personnel, and the results are automatically transmitted for clinical evaluation, thereby enabling an integrated information flow and reducing the time from assessment to clinical decision-making [31].

There is also needed to identify biomarkers associated with increased drug penetration across the blood–labyrinth barrier and with the severity of ototoxicity in the context of inflammatory responses, as well as prognostic, genomic, and diagnostic biomarkers of cochlear injury [15,27].

It is also important to develop methods that enable the identification of early events leading to hair cell death, as well as tools that allow risk prediction based on mechanisms of drug entry and their subcellular localization. Ouyang et al. highlighted the role of MET channels and transporters as determinants of aminoglycoside and cisplatin uptake by hair cells, drawing attention to the rapid advances in structural biology and the application of molecular dynamics simulations to elucidate drug–channel/transporter interactions and to identify potential targets for protective strategies [29,50].

With respect to the development of protective strategies against ototoxicity associated with anticancer treatment, two main trajectories can be distinguished: the search for small-molecule drugs and otoprotective agents, and the refinement of methods for delivering protective substances to the inner ear, such as nanoparticles, hydrogels, or systems activated by environmental stimuli [4,16,28]. Efforts are also directed toward limiting drug (e.g., cisplatin) penetration into the inner ear and into hair cells, ranging from modulation of inflammation and oxidative stress to interventions targeting programmed forms of cell death [19].

An example of an otoprotective strategy is research on the natural compound mangiferin, whose efficacy has been evaluated in cell-based systems, cochlear explants, and animal models. Mangiferin reduced ROS accumulation and inhibited apoptosis via the mitochondrial pathway, while transcriptomic analyses, further validated using RT-qPCR and Western blotting, indicated that modulation of MAPK signaling contributes to the observed protective effect [20]. Another compound with considerable otoprotective potential may be luteolin, which has been shown to inhibit ferroptosis-related processes and to attenuate cisplatin-induced ototoxicity in a murine model [51]. It has also been demonstrated that neomycin inhibits mitophagy dependent on the PINK1–PRKN axis, whereas pharmacological induction of mitophagy with deferiprone and activation of PINK1 with kinetin reduced apoptosis and hair cell loss and were associated with partial preservation of auditory function assessed by ABR in an in vivo model [52]. Research has also addressed pathways involved in auditory cell injury, including the protective effects of caspase inhibitors in cochlear explant models, and has highlighted the potential of pharmacological approaches targeting apoptotic pathways, which have been linked to the preservation of hair cell integrity and protection of auditory function in experimental models [53]. In other studies, inhibition of the HMGB1/RAGE axis was shown to protect against cisplatin-induced ototoxicity by suppressing markers of inflammation and oxidative stress, as assessed in both in vitro and in vivo models using objective measures of hearing as well as histological and biochemical analyses of cochlear tissues [54]. Conversely, Sung et al. applied pharmacological depletion of resident macrophages using a CSF1R receptor inhibitor (PLX3397), reporting a significant reduction in cisplatin-induced hearing loss, improved survival of outer hair cells, and concurrent protection of the kidneys against nephrotoxicity [55].

Another important aspect is the identification of selected candidate biomarkers of cisplatin-induced cochlear injury. One such candidate is the HMGB1/RAGE axis. Cisplatin exposure has been shown to activate this pathway in cochlear hair cells, accompanied by an intensified inflammatory response, oxidative stress, and apoptosis, whereas its inhibition exerts a protective effect both in vitro and in vivo [54]. Another promising candidate is transferrin receptor 1 (TfR1), described as a biomarker of ferroptosis, whose expression increased in outer hair cells following cisplatin administration [51]. At the same time, the rapid development of digital solutions cannot be overlooked. In the future, ototoxicity monitoring may take on a more decentralized form, as tablet-based audiometry performed by appropriately trained non-audiologist personnel may represent one of the most effective approaches to improving access to care. Such solutions enabled screening to be conducted within 72 h of treatment initiation and facilitated earlier referral of patients for comprehensive audiological assessment [31].

The studies described highlight important aspects; however, they should not be interpreted as directly practice-changing. It must be taken into account that most of them are based primarily on in vitro systems, animal models, or tightly controlled experimental conditions, which do not fully reflect the complexity of cancer treatment.

## 6. Conclusions

Ototoxicity represents clinically significant complication of anticancer treatment in children, as even subtle degrees of hearing loss, during developmental periods, may adversely disrupt speech acquisition as well as educational performances and psychosocial functioning. The most robustly documented risk is associated with platinum-based compounds (particularly cisplatin and carboplatin); however, other agents such as aminoglycosides, as well as selected newer therapies (e.g., immunotherapy), are also relevant. The level of risk depends on a range of patient- and treatment-related factors including age, renal function, baseline hearing status, and combined exposures.

Effective protection of the pediatric patient should not rely on a single examination, but rather on the implementation of a standardized hearing monitoring program based on baseline assessment, evaluations during successive treatment cycles, and follow-up after therapy completion, with a preference for methods sensitive to early high-frequency damage (EHF) and for objective measures (DPOAE/ABR) in cases where the child’s cooperation is limited. At the same time, the choice of assessment criteria (ASHA, Brock, SIOP Boston, Chang/CTCAE) has a tangible impact on ototoxicity diagnostics and data comparability, underscoring the need to develop coherent and standardized diagnostic frameworks.

Therefore, children undergoing anticancer therapy with potential ototoxic effects should initially undergo a baseline audiological assessment, followed by monitoring during treatment and careful follow-up after the completion of therapy. Equally important is the appropriate selection of methods tailored to the child’s age and clinical condition. Future research should focus on standardizing monitoring and classification frameworks, validating surveillance models, and determining which biomarkers and otoprotective strategies are sufficiently reproducible, safe, and clinically meaningful for routine use in pediatric oncology practice.

## Figures and Tables

**Table 1:** Ototoxic agents and monitoring strategies.

Agent	Incidence of Ototoxicity	Key Characteristics	Recommended Monitoring
Cisplatin	20–70%	Progressive, bilateral, high-frequency sensorineural hearing loss; typically irreversible	Pure-tone audiometry (PTA), extended high-frequency audiometry (EHF), otoacoustic emissions (OAE), auditory brainstem response (ABR)
Carboplatin	10–30%	Less frequent and less severe ototoxicity; primarily high-frequency involvement	PTA, OAE
Aminoglycosides	20–50%	Cochlear and vestibular toxicity; may occur even after short exposure	OAE, ABR
Immune checkpoint inhibitors	Variable	Rare, heterogeneous audiovestibular manifestations (hearing loss, tinnitus, balance disorders)	Clinical assessment combined with audiological testing

Abbreviations: PTA—pure-tone audiometry; EHF—extended high-frequency audiometry; OAE—otoacoustic emissions; ABR—auditory brainstem response.

## Data Availability

No new data were created or analyzed in this study. Data sharing is not applicable to this article.
